# Application of brain ultrasound in premature infants with brain injury

**DOI:** 10.3389/fneur.2023.1095280

**Published:** 2023-02-13

**Authors:** Lu Liu

**Affiliations:** School of Cyberspace Security, Xi'an University of Posts and Telecommunications, Xi'an, Shaanxi, China

**Keywords:** cranial ultrasound, intracranial hemorrhage, periventricular leukomalacia, hypoxic-ischemic encephalopathy, premature infants

## Abstract

Brain injury is the main factor affecting the development and prognosis of the nervous system in premature infants. Early diagnosis and treatment are of great significance in reducing mortality and disability and improving the prognosis of premature infants. Craniocerebral ultrasound has become an important medical imaging method for evaluating the brain structure of premature infants due to its advantages of being non-invasive, cheap, simple, and bedside dynamic monitoring since it was applied to neonatal clinical practice. This article reviews the application of brain ultrasound to common brain injuries in premature infants.

## 1. Introduction

In recent years, with the continuous improvement of medical levels and the rapid development of neonatal intensive care technology, the rescue success rate of critically ill newborns such as premature infants and very low birth weight infants (VLBW) has been significantly improved. However, due to immature cerebrovascular development and poor cerebral blood flow self-regulation, these types of neonates are prone to cerebral hemodynamic disorders, thus, the incidence of brain injury is usually higher than in term infants (1, 2). The common brain injury in premature infants mainly includes intracranial hemorrhage, periventricular leukomalacia, and hypoxic-ischemic encephalopathy (3). However, these brain diseases do not have significant or specific clinical symptoms at an early stage, thus, it is necessary to use safe, reliable, and convenient imaging examinations for auxiliary diagnosis. As a simple, convenient, and non-invasive examination method (4, 5), brain ultrasound plays an important role in the early diagnosis, severity judgment, and prognosis evaluation of brain injury in premature infants. This article reviews the application of brain ultrasound to common brain injuries in premature infants.

## 2. Characteristics and advantages of craniocerebral ultrasound

Brain ultrasound is a technique that uses specific sound waves to understand the structure and pathological changes of brain tissue (6). At present, there are two common methods of brain ultrasound, namely, B-mode ultrasound and color Doppler ultrasound (7). B-mode ultrasound belongs to two-dimensional imaging. Real-time imaging is performed on the screen in grayscale. We can clearly observe the intracranial structure and brain center lesions by sector scan (8). Color Doppler ultrasound in the B-mode ultrasound on the basis of increased blood flow, can effectively show changes in brain lesions and lesion tissue blood flow velocity, vascular distribution, resistance index, and other hemodynamic signals (9), can more clearly reflect the severity of the disease.

The emergence of brain ultrasound technology has opened up a new way for clinicians to understand intracranial lesions *in vivo* (6). This technology has the advantages of simple and rapid operation, high accuracy, non-invasive, no radiation, intuitive reflection, and bedside dynamic monitoring and has become the first choice for the clinical diagnosis of brain injury (10).

## 3. Methods and timing of cranial ultrasound examination

### 3.1. Methods of cranial ultrasound examination

Brain ultrasonography should be performed according to specific procedures. First, select the appropriate scanning probe, set the corresponding probe frequency, and disinfect the probe; then, the children are placed in this position, usually supine or prone, to ensure that the children are in a quiet state and usually do not take sedatives; after that, the examiner is located on the right or top of the patient, and the patient is continuously scanned from front to back, from center to both sides on the coronal and sagittal planes, through the deflection probe. During the examination, attention should be paid to the continuity of the scan to prevent missed diagnoses.

According to the different scanning positions, craniocerebral ultrasound can be divided into four parts, namely, anterior fontanel scanning, lateral fontanel scanning, mastoid fontanel scanning, and posterior fontanel scanning. Among them, the anterior fontanelle is the preferred inspection site (11, 12). During scanning, attention should be paid to comparing the parenchymal structure and echo of the two cerebral hemispheres and observing the changes in the shape and position of the ventricle and midline. Scans of the posterior fontanelle reveal brain structures close to the horizontal position, which can be used to compensate for the lack of detection of acoustic images of the bottom of the brain when scanning the anterior fontanelle. Transcranial fontanel scanning, which is equivalent to observing a cross-sectional view of the brain from one side, is often used as an acoustic window for cerebrovascular hemodynamics. The mastoid fontanelle can be used to observe the posterior fossa. During the scan, attention should be paid to the boundary between the cerebellar hemisphere, cerebellar vermis, cortex and medulla, material echoes, and changes in the cerebellar medullary cistern (13).

### 3.2. Time of cranial ultrasound examination

Brain ultrasound is used to diagnose intracranial structures and lesions through the unclosed salt gate. After birth, the salt door will gradually close, thus, it is necessary to complete the craniocerebral ultrasound examination within a specific time period (14–16). Usually, the first cranial ultrasound examination is performed within 3 days after birth, no later than 1 week. For infants with a history of hypoxia, asphyxia, and very low birth weight (VLBW), the first brain ultrasound examination should be performed within 24–48 h after birth to initially assess the brain condition. Thereafter, a review should be conducted every other week until the full moon. If necessary, a 3–6 months follow-up review can be performed.

## 4. Diagnostic value of brain ultrasound in premature infants with brain injury

### 4.1. Ultrasonic diagnosis of intracranial hemorrhage in premature infants

Premature intracranial hemorrhage is one of the most common pediatric diseases. The majority occurred within 3 days after birth. The cause of the disease is usually perinatal hypoxia, which is harmful. According to the study of Sawyer et al. (17), the mortality rate caused by intracranial hemorrhage is as high as 50% in premature infants with a gestational age of fewer than 32 weeks and a weight <1,500 g. However, in the early stages of cerebral hemorrhage, children usually do not show obvious clinical symptoms, and we can only use imaging tools to diagnose. In many imaging methods, craniocerebral ultrasound has the advantages of non-invasive, high repeatability, bedside rapid, and effective evaluation of the evolution of cerebral hemorrhage, which makes it the first choice for the diagnosis of a cerebral hemorrhage in premature infants (18). Yue Xiangzhu can provide an imaging basis for clinical treatment.

**Figure 1:**
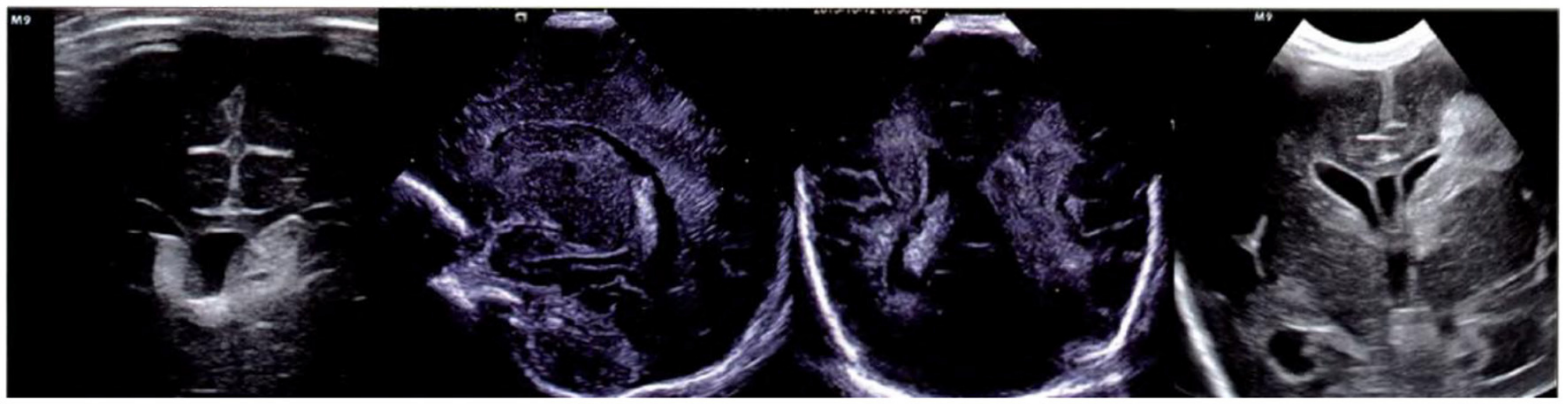
Periventricular intraventricular hemorrhage (Papile classification) (20).

According to the different bleeding sites, cerebral hemorrhage in premature infants can be divided into a periventricular intraventricular hemorrhage, cerebral hemorrhage, subdural hemorrhage, and subarachnoid hemorrhage. (1) Ultrasonic diagnosis of periventricular intraventricular hemorrhage: In 1978, Papile et al. (19) performed computed tomography on very low birth weight infants (VLBW). According to the amount of intraventricular hemorrhage, periventricular intraventricular hemorrhage is divided into four levels. Grade I: bleeding is limited to the germinal base. Ultrasonography showed a large amount of high echo in the anterior horn of the lateral ventricle in the coronal plane and caudate nucleus sulcus in the sagittal plane. Grade II: intraventricular hemorrhage occurred, but the ventricle was not dilated. Ultrasonographic manifestations were lateral ventricle or choroid plexus patchy or massive high echo. Grade III: intraventricular hemorrhage with ventricular dilatation. Ultrasonography showed that the lateral ventricle was partially or completely filled with a large amount of hyperechoic blood and would expand. Grade IV: intraventricular hemorrhage with periventricular hemorrhagic cerebral infarction; ultrasonography revealed a sectorial or spherical hyperechoic zone in the periventricular white matter. Figure 1 shows the ultrasound images of periventricular intraventricular hemorrhage at all levels, and the hemorrhage is classified from left to right as grades I–IV (20). (2) Ultrasound diagnosis of cerebral hemorrhage: In the early stage of hemorrhage, there was a strong echo mass in the brain parenchyma with a clear boundary. With the development of the disease, the old hematoma gradually liquefied absorption, and brain parenchyma has a weak echo or a small echo-free zone. (3) Ultrasound diagnosis of subdural hemorrhage: there is a prominent crescent-shaped, narrow, strong echo between the skull and brain tissue. With the decrease in the amount of bleeding in the later period, the echo will gradually change into a narrow silent space. (4) Ultrasound diagnosis of subarachnoid hemorrhage usually occurs at the edge of the brain. A large number of studies (21–23) have shown that brain ultrasound is less sensitive to identify these areas of bleeding, easy to missed diagnosis, which is the weak link of brain ultrasound diagnosis.

### 4.2. Ultrasonographic diagnosis of periventricular leukomalacia in premature infants

Periventricular leukomalacia is a disease closely related to motor function, audiovisual function, and cognitive function. It is the most serious case of brain injury in premature infants and one of the important causes of neonatal cerebral palsy (24). Li Jinhui et al. (25) showed that periventricular leukomalacia is related to the following three interactive factors: (1) the development of cerebral white matter vessels in premature infants is not perfect; (2) immature cerebral blood flow autoregulation function; and (3) white matter oligodendrocytes and their precursors are very fragile; the smaller the gestational age, the higher the incidence of periventricular leukomalacia. Most premature infants with periventricular leukomalacia have no obvious clinical symptoms, and the diagnosis usually depends on an imaging examination. Magnetic resonance imaging (MRI) is the best method to diagnose periventricular leukomalacia. However, MRI examination requires moving children, which takes a long time and has limited application value for critically ill children. A brain ultrasound examination can not only be completed at the bedside but also involve real-time observation of the brain condition in order to facilitate a timely understanding of the development of the disease. In recent years, with the rapid development of ultrasound equipment and technology, the accuracy, sensitivity, and specificity of brain ultrasound for periventricular leukomalacia reached 91, 100, and 33%, respectively (26), which has become the main means of diagnosis of periventricular leukomalacia (27).

**Figure 2:**
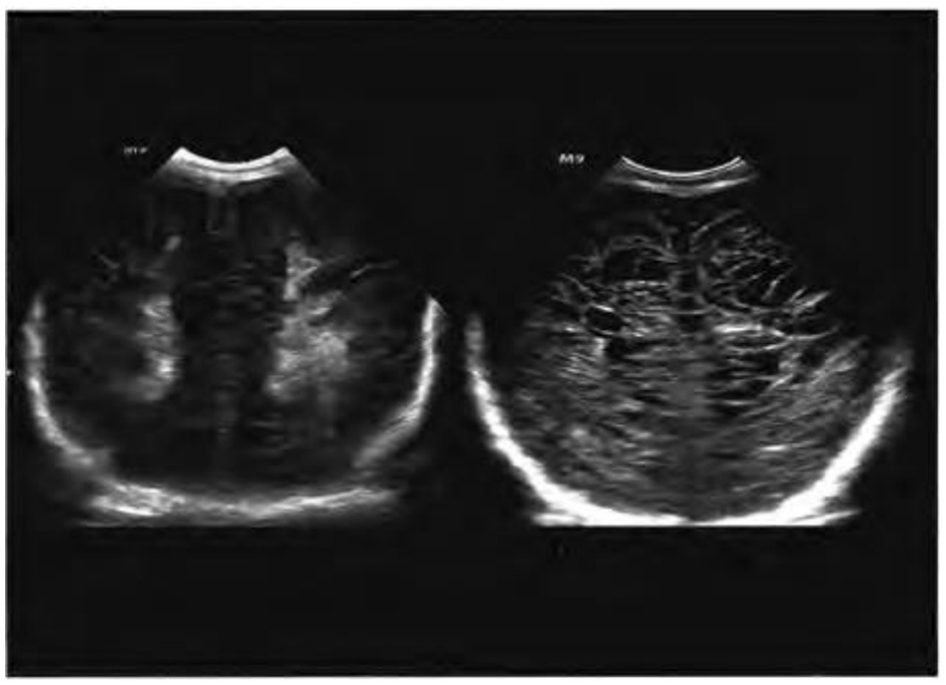
Ultrasonography of periventricular leukomalacia in the same part of the same child (20).

At present, according to different periods of periventricular leukomalacia ultrasound performance, it is generally divided into four levels (28). Grade I: bilateral periventricular white matter echo-transient enhancement (1, 29), duration of more than a week. This is a reversible disease with an ideal prognosis. When diagnosing this degree of periventricular leukomalacia, attention should be paid to distinguishing white matter echoes from those of normal preterm infants. In general, the echo of the former is rough and uneven, while the echo of the latter is thin and uniform without an obvious boundary. Grade II: strong echo around the ventricles gradually evolves into a local small cyst. The location and extent of cysts are key to predicting the prognosis. According to the study of Gotardo et al. (30), cysts located around the central sulcus may lead to bilateral spastic cerebral palsy, while cysts located in the frontal lobe are usually not related to cerebral palsy. Grade III: multiple cysts of the forehead and occipital white matter. Grade IV: multiple subcortical cysts formed in the deep white matter hyperechoic area around the ventricle. Studies (31) have shown that periventricular leukomalacia occurs in children with a cyst formation time of 4 weeks after birth. When MRI is used at term, it usually shows that the lesion has been absorbed. Therefore, in the diagnosis of periventricular leukomalacia, it is necessary to continuously use brain ultrasound scans to track and observe the changes in cysts within 4–6 weeks after birth. Figure 2 shows the sonogram of different degrees of periventricular leukomalacia in the same part of the same patient, in which the echo of bilateral occipital white matter in the left figure was significantly enhanced; the right image shows multiple vesicles of different sizes (20).

### 4.3. Ultrasonographic diagnosis of hypoxic-ischemic encephalopathy in premature infants

Neonatal hypoxic-ischemic encephalopathy is hypoxic-ischemic brain damage caused by perinatal hypoxia and asphyxia. It is one of the most common brain injuries in premature infants and one of the leading causes of neonatal neurodevelopmental abnormalities and death (32). According to the statistics of Cai Qing et al. (33), the incidence of neonatal hypoxic-ischemic encephalopathy is 1/1,000–1/2,000 live full-term infants, of which 15–20% died in the neonatal period, 25–30% of survivors have permanent neurological deficits, such as cerebral palsy and mental disability. Early diagnosis and intervention are important ways to reduce brain injury and improve the prognosis for children. However, as early neonatal hypoxic ischemic encephalopathy usually has no obvious clinical symptoms, imaging methods must rely on further examination and diagnosis. Craniocerebral ultrasound can not only dynamically observe the two-dimensional structure of the brain but also provide help for the classification of neonatal hypoxic-ischemic encephalopathy. It can also obtain cerebral hemodynamic parameters through color Doppler technology, providing a richer diagnostic basis for clinical diagnosis. Compared with brain CT and MRI, brain ultrasound can detect abnormal changes in brain time, which is of great significance for the early diagnosis and treatment of neonatal hypoxic-ischemic encephalopathy.

The brain ultrasound diagnosis of hypoxic ischemic encephalopathy is based on the pathological process of the disease. In the early stages of the disease, the main pathological change is brain edema. Ultrasound showed diffuse and uneven echo enhancement in both cerebral hemispheres, with the most obvious enhancement in the periventricular white matter, even equal to the intensity of the choroid plexus. In the late stages of the disease, cystic or atrophic changes occur in the brain structure, and the ultrasound images can be clearly seen after 3–4 weeks. Cystic changes usually occur after extensive and severe brain edema. Ultrasound showed cysts of different sizes in the original echo-enhanced area. Atrophic changes are secondary to extensive brain injury. Ultrasound examination showed the extensive division of the brain, deep sulci, widened extra-frontal space, and atrophy of the gyrus (34).

Pathological changes of neonatal hypoxic ischemic encephalopathy are a dynamic evolution process, mainly based on cerebral blood. Blood flow changes can be used for diagnosis. Color Doppler ultrasound can directly reflect the blood perfusion of lesions through the changes in the blood flow spectrum (35). By comparing the cerebral blood flow of 38 neonates with hypoxic-ischemic encephalopathy and 30 full-term healthy neonates, Yang Guanghui found that within 48 h after hemorrhage, the peak systolic velocity (Vs), end diastolic velocity (Vd), resistance index (RI), and pulsatility index (PI) of children with mild hypoxic-ischemic encephalopathy were close to those of the control group. The Vs and Vd of children with moderate and severe hypoxic-ischemic encephalopathy were significantly lower than those of the control group, while RI and PI were significantly higher than those of the control group, and the difference was *P* < 0.05. The follow-up study of Guan (36) and Liao Huifang (37) was similar to theirs. It is suggested that color Doppler ultrasound detection of neonatal hypoxic-ischemic encephalopathy can effectively understand the changes in cerebral hemodynamics in children and has important reference significance for predicting and pre-evaluating the degree of brain injury in children. In the previous content, relevant studies have demonstrated the value of craniocerebral ultrasound in the diagnosis of neonatal symptoms (38, 39).

## 5. Conclusion

In conclusion, craniocerebral ultrasound, as a non-invasive, cheap, simple, and bedside brain medical imaging method, can be used to screen and diagnose intracranial lesions in infants who have not yet closed the halo. Especially in the diagnosis of brain injury in premature infants, brain ultrasound shows good accuracy and specificity, which can clearly show the condition of brain injury and the hemodynamic changes of diseased tissues. Therefore, as an important screening project for brain injury in premature infants, it is conducive to the early detection of intracranial lesions in children and can provide an accurate basis for early clinical diagnosis and prognosis evaluation. In recent years, with the continuous improvement of brain ultrasound imaging, brain ultrasound has become the preferred diagnosis of brain disease in premature infants.

## Author contributions

The author confirms being the sole contributor of this work and has approved it for publication.
